# Is there a linear relationship between the dose of ruminant *trans*-fatty acids and cardiovascular risk markers in healthy subjects: results from a systematic review and meta-regression of randomised clinical trials

**DOI:** 10.1017/S0007114514002578

**Published:** 2014-10-27

**Authors:** Constance Gayet-Boyer, Fanny Tenenhaus-Aziza, Caroline Prunet, Corinne Marmonier, Corinne Malpuech-Brugère, Benoit Lamarche, Jean-Michel Chardigny

**Affiliations:** 1 CNIEL, 42 rue de Châteaudun, 75009Paris, France; 2 Clermont Université, Université d'Auvergne, UNH, BP 10448, F-63000Clermont-Ferrand, France; 3 INRA, UMR 1019, UNH, CRNH Auvergne, F-63000Clermont-Ferrand, France; 4 Institute of Nutraceuticals and Functional Foods, Laval University, Québec, QC, Canada

**Keywords:** Ruminant *trans*-fats, Cardiovascular markers, Randomised clinical trials, Systematic review

## Abstract

The effects of ruminant (R) *trans*-fatty acids (TFA) on the risk of CVD are still under debate. It could be argued that the lack of the effect of R-TFA may be the result of the small amount of their intake. Taking into consideration the growing available data from intervention studies, we carried out a systematic review and meta-regression to assess the impact of R-TFA intake levels on changes in the total cholesterol: HDL-cholesterol (TC:HDL-C) ratio. A systematic review of the literature was conducted and thirteen randomised clinical trials were included, yielding a total of twenty-three independent experimental groups of subjects. A univariate random-effects meta-regression approach was used to quantify the relationship between the dose of R-TFA and changes in the TC:HDL-C ratio. To consider several potential modifiers such as subject and dietary characteristics, a multivariate regression analysis was performed. We found no relationship between R-TFA intake levels of up to 4·19 % of daily energy intake (EI) and changes in cardiovascular risk factors such as TC:HDL-C and LDL-cholesterol (LDL-C):HDL-C ratios. In addition, a multivariate regression analysis that included other dietary variables, as well as subject baseline characteristics, confirmed that doses of R-TFA did not significantly influence the changes in the lipid ratio. Our findings showed that doses of R-TFA did not influence the changes in the ratios of plasma TC:HDL-C and LDL-C:HDL-C. These data suggest that TFA from natural sources, at least at the current levels of intake and up to 4·19 % EI, have no adverse effects on these key CVD risk markers in healthy people.

Since the 1990s, *trans*-fatty acids (TFA) have been linked to harmful effects, as they induce not only an increase in LDL-cholesterol (LDL-C) concentration but also a decrease in HDL-cholesterol (HDL-C) concentration^(^ ^1^ ^)^. Besides detrimental health implications on lipid metabolism, they also have a plethora of other undesirable cardiometabolic effects including pro-inflammatory effects and endothelial dysfunction. The generic term of TFA represents two independent dietary sources, i.e. an industrial one (industrially produced *trans*-fatty acids; IP-TFA) and a natural counterpart (ruminant *trans*-fatty acids; R-TFA). In 1993, Willet *et al.* ^(^ ^2^ ^)^ underlined the difference in the impact of both dietary sources in the Nurses’ Health Study, but the first intervention studies were only published in 2008^(^ ^3^ ^,^ ^4^ ^)^.

IP-TFA and R-TFA differ greatly in their isomer distribution (i.e. the relative distribution of the position of the *trans* double bond along the carbon chain) as well as in their prevalence in food sources. R-TFA are generally present in food at low levels (up to 8 % of total fatty acids in milk fat), whereas IP-TFA may reach up to 61 % of total fatty acids in pastries and shortenings^(^ ^5^ ^)^. In the past decade, the quantities of IP-TFA have been dramatically reduced in numerous food products, but they remain present according to specific recipes or dietary habits^(^ ^5^ ^)^.

Several well-conducted studies in animal models have suggested that R-TFA and IP-TFA have different impacts on CVD risk markers. Most of them have not supported the adverse effects of R-TFA on atherosclerosis and risk factors of CVD. For example, two different studies from Wang *et al.* ^(^ ^6^ ^,^ ^7^ ^)^ carried out in a rodent model of dyslipidaemia (JCR:LA-cp rats) showed either neutral or beneficial effects of a vaccenic acid-supplemented diet on the plasma concentrations of total cholesterol (TC), LDL-C and fasting and postprandial TAG. A considerable amount of other studies conducted in animal models focused on the investigation of the effect of dietary conjugated linoleic acid (CLA) supplementation, either a mixture of isomers or specific ruminant CLA (*c*9, *t*11-CLA). When fed with diets rich in *c*9, *t*11-CLA, animals showed an improved lipoprotein profile and/or reduced lesion development, suggesting a beneficial effect of the ruminant CLA isomer on atherosclerosis^(^ ^8^ ^,^ ^9^ ^)^. Very few clinical trials have studied the effects of R-TFA on the risk factors of CVD, and they have reported conflicting results. A highly controlled study by Tholstrup *et al.* ^(^ ^10^ ^)^ investigated the effect of R-TFA-enriched butter intake on lipid and lipoprotein profiles. They showed a decrease in TC and HDL-C concentrations, but no changes in plasma LDL-C concentration and the TC:HDL-C ratio compared with the control. Chardigny *et al.* ^(^ ^3^ ^)^ found that in women, an R-TFA diet increased the concentrations of LDL-C and HDL-C compared with a diet supplemented with IP-TFA. As a result, there was no significant modification in the diagnostic ratios of TC:HDL-C and of apoA1:apoB. Motard-Bélanger *et al.* ^(^ ^4^ ^)^ investigated the effects of R-TFA at various doses. They showed that a moderate R-TFA intake had no impact on the plasma concentrations of either LDL-C or HDL-C, or on the ratio of TC:HDL-C. They also showed that a high R-TFA intake significantly reduced the concentrations of HDL-C and increased the concentrations of TC and LDL-C, and the ratio of TC:HDL-C. Similar results were obtained when computing the ratio of LDL-C:HDL-C. A recent study by Lacroix *et al.* ^(^ ^11^ ^)^ reported no significant effect of an R-TFA diet on the concentrations of TC and LDL-C compared with a control diet. However, they reported that an increase in R-TFA intake may slightly lower the concentrations of HDL-C. Such results suggest that a diet rich in R-TFA may influence the changes in the concentrations of HDL-C, which emphasises the importance of considering the lipid ratios of TC:HDL-C and LDL-C:HDL-C as the surrogates of CVD risk in dietary studies. The independent relevance of HDL-C in the assessment of cardiovascular-related mortality has been demonstrated, and it is now established that the effects of dietary fat on the TC:HDL-C ratio may differ from their effects on LDL-C concentrations^(^ ^12^ ^)^.

Previous epidemiological studies have demonstrated the lack of any adverse effect of R-TFA intake on the risk of CHD^(^ ^2^ ^,^ ^13^ ^)^, supported by the data of a meta-analysis of cohort studies reported by Bendsen *et al.* ^(^ ^14^ ^)^. A recent prospective study has confirmed the adverse effects of IP-TFA intake on cardiovascular health, whereas the negative impact of R-TFA intake on cardiovascular health has been found to be no longer significant after additional adjustment^(^ ^15^ ^)^. However, these published prospective cohort studies can be susceptible to residual confounding factors such as difficulties in assessing dietary intakes.

The latest review from the literature has shown evidence that further research is needed on the specific effects of R-TFA on the risk of CVD^(^ ^16^ ^)^. One major question that remains unclear is whether the lack of any effect of R-TFA is a result of the small amount of their intake. It could also be argued that a threshold may exist and a low intake of R-TFA may even have beneficial effects. Hansen *et al.* ^(^ ^17^ ^)^ recently demonstrated an inverse association between lower intakes of R-TFA and change in body weight.

To achieve a more exhaustive estimation of the effects of R-TFA intake, we first performed a systematic review. We then analysed the data from thirteen randomised clinical trials to quantify the relationship between the dose of R-TFA and the change in the ratios of TC:HDL-C and LDL-C:HDL-C in healthy adults.

## Methods

### Study selection

A systematic literature search was conducted on studies published between January 1975 and December 2011, using the following search terms on the PubMed and Scopus databases, respectively: ((‘Fatty Acids/blood’ OR ‘Cholesterol/blood’ OR ‘Lipoproteins/blood’) AND (‘Dairy Products’ OR ‘Trans Fatty Acids’ OR ‘Linoleic Acids, Conjugated’)) and: ((‘fatty acids’) OR cholesterol OR lipoproteins) AND ((‘dairy products’) OR (‘trans fatty acids’) OR (‘linoleic acids, conjugated’)) AND ((clinical trial) OR (controlled study)). An updated secondary search was conducted on studies published up until December 2013. The search strategy had language (English) and study design (clinical trial, randomised controlled trial) restrictions. We also limited our search to studies conducted in adults. The following inclusion criteria were established: healthy volunteers; dairy products as the primary source of fat in the experimental diets; clear reporting and documentation of the amounts of R-TFA consumed; nil or negligible amounts of IP-TFA in the diet; feeding period >3 weeks; availability of data on blood lipids. The selection process was conducted by two investigators (C. G.-B. and J.-M. C.).

### Data extraction and classification

From each study, we extracted quantitative data that were adjusted according to the measurement unit, and all the relevant information on (1) study characteristics such as first author, year of publication, study design and country of origin, (2) subject characteristics such as name of the group (group name), sample size (*n*), sex, age and BMI, (3) lipid and lipoprotein blood concentrations at baseline and at the end of the intervention period such as TC, LDL-C, HDL-C, TAG in mmol/l, and (4) diet characteristics including total energy intake (EI) in MJ, daily energy from carbohydrate (CARB), protein (PROT) and fat (FAT) in percentage of EI, fatty acid composition of experimental diets in percentage of EI, such as SFA, MUFA, PUFA, total R-TFA, total R-*trans*-18 : 1 (total R-18 : 1*t*), vaccenic acid (*trans*-11 18 : 1), rumenic acid (*cis*-9, *trans*-11 18 : 2) and total CLA. Where such data were not provided, requests were made to investigators. When data were available, they were separated by sex. Where studies provided data for two or more R-TFA-treated groups, they were included as separate and independent estimates in the analysis.

### Determination of ruminant trans-fatty acid intake

For the purpose of the codex guidelines on nutrition labelling and other codex-related standards and guidelines, TFA (IP-TFA or R-TFA) are defined as all the geometrical isomers of MUFA and PUFA having non-conjugated, carbon–carbon double bonds in the *trans* configuration^(^ ^18^ ^)^. With regard to dairy fat composition, it has been reported that total R-18 : 1*t* represents approximately 80 % of the total R-TFA^(^ ^19^ ^)^, and most trials have evaluated the effects of 18 : 1 TFA. Therefore, in the present study, we used R-18 : 1*t* data to estimate whether intake levels of R-TFA would be associated with a change in the ratios of TC:HDL-C and LDL-C:HDL-C. When R-18 : 1*t* data were not available^(^ ^20^ ^)^, the amounts of total R-18 : 1*t* in the diet were assessed based on the total R-TFA intake data.

### Definition of study outcomes

Prior studies have shown that the ratio of TC:HDL-C is twice as informative of the individual risk of cardiovascular death as TC or LDL-C concentration^(^ ^21^ ^)^. The TC:HDL-C ratio reflects changes in both LDL and HDL concentrations, and differences in this ratio within and among populations are predominantly due to lifestyle factors such as diet, obesity and physical activity^(^ ^21^ ^)^. To date, the ratio of TC:HDL-C is probably the most robust lipid metric to estimate lifestyle factor-related CVD risk. However, the ratio of LDL-C:HDL-C is an indicator commonly used to estimate the risk of CVD. In our approach, each group of subjects acted as their own control. Thus, based on the extracted data, changes in the ratios of both TC:HDL-C (Δ_TC:HDL-C_) and LDL-C:HDL-C (Δ_LDL-C:HDL-C_) between the end of the intervention and baseline were calculated. For the sake of uniformity, we recalculated Δ_TC:HDL-C_ and Δ_LDL-C:HDL-C_ from mean TC, LDL-C and HDL-C levels for all studies. Standard deviations of change from the baseline to the endpoint were extracted when provided, and imputed for the article with missing standard deviations by using the method referenced in the Cochrane Handbook for Systematic Reviews of Interventions^(^ ^22^ ^)^.

### Statistical analysis

To test whether there is a linear relationship between the total R-18 : 1*t* intake levels and Δ_TC:HDL-C_ or Δ_LDL-C:HDL-C_, we used the univariate random-effects meta-regression approach^(^ ^23^ ^)^. To consider both within-trial variances of treatment effect and the residual between-trial heterogeneity, we used weighted least-squares regressions. The weighting factor was defined as 1/(σ^2^+τ^2^), where σ^2^ is the variance within a study and τ^2^ is the variance between studies.

To explore environmental factors that might influence the changes in the ratios of TC:HDL-C and LDL-C:HDL-C next to R-18 : 1*t* intake (such as subject and study characteristics), two backward stepwise partial least-squares (PLS) regressions were implemented.

The independent variables of each model were as follows: TC; HDL-C; LDL-C; TAG; age; BMI (baseline values); SFA; MUFA; PUFA; CARB; FAT; PROT; total R-18 : 1*t*; EI (values related to the intervention diet). Sex as an independent variable was not included because of a lack of data (six among the twenty-three experimental groups were mixed). Compared with classical multivariate regression, PLS regression enabled correlated variables and observations containing missing data^(^ ^3^ ^)^ to be taken into account. PLS regression also took into account the fact that we had an important number of independent variables (fourteen variables). The results of PLS regression using the selected independent variables were extracted for each model (*R* ^2^, parameter coefficients of the final model and CI). Statistical analyses were performed using R software (metafor package; R Core Team), SAS statistical software (version 9.2; SAS Institute) and Simca-P+ software (version 12.0.1; Umetrics). The significance level was set at the 5 % level.

## Results

### Study selection and data extracted

**Fig. 1:**
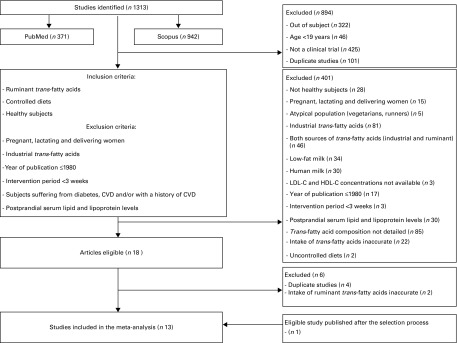
Flow chart for the selection of eligible studies. LDL-C, LDL-cholesterol; HDL-C, HDL-cholesterol.

The initial search allowed us to identify 1313 studies (371 and 942 from the PubMed and Scopus databases, respectively), of which twelve were selected as appropriate for inclusion in the present meta-analysis^(^ ^3^ ^,^ ^4^ ^,^ ^10^ ^,^ ^20^ ^,^ ^24^ ^–^ ^31^ ^)^. In addition, one recently published study^(^ ^11^ ^)^ was included after the selection process. Altogether, the thirteen trials that met our criteria yielded twenty-three experimental groups of subjects included as independent data points in the present meta-analysis. We included all the groups of subjects when found to be eligible with respect to the criteria of selection, although the first goal of the clinical trial they were part of was not to assess specifically the effect of R-TFA^(^ ^20^ ^,^ ^29^ ^,^ ^30^ ^)^. Data were derived from 666 volunteers. The trial flow is summarised in Fig. 1.

The data extracted from the thirteen studies included in the present meta-regression are shown in Tables 1 and 2. All the participants were healthy. The mean baseline BMI ranged from 21·1 to 31·2 kg/m^2^. Of the thirteen trials, seven enrolled participants with a normal body weight and six included overweight or obese subjects. The mean baseline TC:HDL-C ratio varied from 2·46 to 5·63 mmol/l. We confirmed homogeneity between the TC:HDL-C values at baseline among the studies using the metafor package (data not shown). The total EI varied from 7·9 to 14 MJ/d and the duration of the intervention lasted from 3 to 7 weeks. The intake of total R-18 : 1*t* varied quite extensively between the studies, ranging from 0·12 to 4·19 % daily EI (Table 3). The fat in the intervention diets was mainly dairy fat, resulting in R-TFA as the only source of TFA ingested during the experimental period. Studies were conducted in Europe (*n* 7), Canada (*n* 4) and in the USA (*n* 2). To assess study quality, we used the Jadad score, which showed that only two of the thirteen studies were defined as poor quality (Jadad score < 3) (see online supplementary Table S1).

**Table 1:** Composition of the intervention diets, including doses of ruminant-18 : 1 *trans*-fatty acids for the twenty-three experimental groups of subjects, derived from the thirteen eligible randomised clinical trials

Study identification	Experimental group*	Total R-18 : 1*t* (% EI)	Total EI (MJ)	Total fat (% EI)	Carbohydrate (% EI)	Protein (% EI)	SFA (% EI)	PUFA (% EI)	MUFA (% EI)
Wood^(^ ^29^ ^)^ (1993)	Butter	2·00	11	39·3	45·1	15·7	21·5	4·1	12·0
Almendingen^(^ ^31^ ^)^ (1995)	Butter	0·50	10	34·8	49·8	15·4	15·7	5·9	9·1
Aro^(^ ^30^ ^)^ (1997)	Run-in	0·80	10·8	32·2	52·0	14·8	13·8	3·4	12·2
	Run-in	0·80	10·8	32·2	52·0	14·8	13·8	3·4	12·2
Tholstrup^(^ ^24^ ^)^ (1998)	Danish milk	0·12	14†	41·5	49·2	11·3	26·0	5·5	8·1
	Milk fat	1·66	14†	40·5	47·7	11·6	20·3	4·8	12·3
Lichtenstein^(^ ^20^ ^)^ (2003)	Butter	1·00	11·7†	29·1	53·9	16·9	16·7	2·4	8·0
	Butter	1·00	8·8†	29·1	53·9	16·9	16·7	2·4	8·0
Biong^(^ ^26^ ^)^ (2004)	Cheese	0·68	8·5	26·2	48·5	25·3	15·2	2·6	7·2
Desroches^(^ ^28^ ^)^ (2005)	Control	0·25	12·3	40·9	46·4	15·3	19·2	7·9	10·6
	CLA	1·69	12·3	40·9	46·4	15·3	19·2	8·0	10·6
Tholstrup^(^ ^10^ ^)^ (2006)	Control	0·32	13·7	41·8	44·9	9·8	24·4	3·6	9·6
	Vaccenic acid	2·14	12·7	44·6	42·1	9·4	22·5	3·8	14·9
Tricon^(^ ^27^ ^)^ (2006)	Control	0·27	10·7	37·9	45·3	16·8	12·5	5·1	9·1
	CLA	2·10	11·4	38·1	45·4	16·5	11·3	4·0	8·0
Chardigny^(^ ^3^ ^)^ (2008)	R-TFA	4·19	9·0	36·0	46·0	15·0	16·1	NA	NA
	R-TFA	4·19	7·9	40·0	45·0	15·0	17·9	NA	NA
Motard-Bélanger^(^ ^4^ ^)^ (2008)	Moderate	1·45	13·6	37·4	49·7	14·0	17·9	4·3	11·7
	High	3·50	13·5	38·1	48·8	14·0	19·0	3·4	9·9
Malpuech-Brugère^(^ ^25^ ^)^ (2010)	L0	0·61	8·8	37·7	47·4	14·9	21·3	3·6	11·8
	L4	0·87	8·6	38·7	44·9	15·4	19·9	3·9	14·0
	L9	2·42	8·4	38·8	46·7	14·5	18·1	5·2	14·3
Lacroix^(^ ^11^ ^)^ (2012)	R-TFA	1·40	9·5	33·0	54·3	15·0	10·3	5·8	12·8

% EI, percentage of energy intake; CLA, conjugated linoleic acid; NA, not available; R-TFA, ruminant *trans-*fatty acid; L0, experimental dairy fat containing 2·9 g R-TFA for 100 g fatty acids; L4, experimental dairy fat containing 4·1 g R-TFA for 100 g fatty acids; L9, experimental dairy fat containing 12·2 g R-TFA for 100 g fatty acids.

* Experimental group, as named in the original articles.

† Data were imputed based on values from pre-intervention diets.

**Table 2:** Mean baseline characteristics of the twenty-three groups of subjects included in the meta-analysis

Study identification	Experimental group*	Baseline TAG (mmol)	Baseline TC (mmol)	Baseline LDL-C (mmol)	Baseline HDL-C (mmol)	Age (years)	BMI (kg/m^2^)
Wood^(^ ^29^ ^)^ (1993)	Butter	1·24	5·23	3·60	1·17	42	25·1
Almendingen^(^ ^31^ ^)^ (1995)	Butter	1·60	5·35	3·58	1·09	28	26·0
Aro^(^ ^30^ ^)^ (1997)	Run-in	1·07	5·42†	3·13†	1·60†	29†	22·9†
	Run-in	1·01	5·29†	3·20†	1·46†	29†	22·9†
Tholstrup^(^ ^24^ ^)^ (1998)	Danish milk	0·85	4·01	2·67	1·15	25	24·0
	Milk fat	0·85	4·01	2·67	1·15	25	24·0
Lichtenstein^(^ ^20^ ^)^ (2003)	Butter	1·56	6·14	4·33	1·09	60	28·1
	Butter	1·79	6·55	4·33	1·37	67	26·6
Biong^(^ ^26^ ^)^ (2004)	Cheese	1·07	5·55†	3·61†	1·47†	31·5†	27·0†
Desroches^(^ ^28^ ^)^ (2005)	Control	1·75	4·85	3·17	1·06	36·6	31·2
	CLA	1·56	4·76	3·11	1·11	36·6	31·2
Tholstrup^(^ ^10^ ^)^ (2006)	Control	0·89	4·87	2·67	1·54	26·1	22·5
	Vaccenic acid	0·88	4·02	2·67	1·32	25·2	23
Tricon^(^ ^27^ ^)^ (2006)	Control	1·09	4·50	2·93	1·09	45·5	25·0
	CLA	1·10	4·46	2·90	1·10	45·5	25·0
Chardigny^(^ ^3^ ^)^ (2008)	R-TFA	0·88	4·38	2·38	1·60	27·7	22·9
	R-TFA	0·93	5·07	2·58	2·06	27·5	21·1
Motard-Bélanger^(^ ^4^ ^)^ (2008)	Moderate	1·14	4·32	2·56	1·25	32·8	23·6
	High	1·14	4·32	2·56	1·25	32·8	23·6
Malpuech-Brugère^(^ ^25^ ^)^ (2010)	L0	0·85	4·42†	2·34†	1·69†	26†	21·7†
	L4	1·08	4·88†	2·46†	1·76†	25†	22·0†
	L9	0·82	4·52†	2·35†	1·62†	28†	21·9†
Lacroix^(^ ^11^ ^)^ (2012)	R-TFA	1·04	5·11	2·84	1·78	38·1	23·6

TC, total cholesterol; LDL-C, LDL-cholesterol; HDL-C, HDL-cholesterol; CLA, conjugated linoleic acid; TFA, *trans*-fatty acid; R-TFA, ruminant *trans*-fatty acid; L0, experimental dairy fat containing 2·9 g R-TFA for 100 g fatty acids; L4, experimental dairy fat containing 4·1 g R-TFA for 100 g fatty acids; L9, experimental dairy fat containing 12·2 g R-TFA for 100 g fatty acids.

* Experimental group, as named in the original articles.

† Mean values for male and female data, as available in the original articles.

**Table 3:** Mean values of post- *v*. pre-diet changes in both ratios of total cholesterol (TC):HDL-cholesterol (HDL-C) and LDL-cholesterol (LDL-C):HDL-C *v*. doses of ruminant-18 : 1 *trans*-fatty acids in the diet for the twenty-three experimental groups of subjects

Study identification	Experimental group*	Total R-18 : 1*t* (% EI)	Total R-18 : 1*t* (g/d)	TC:HDL-C change	LDL-C:HDL-C change
Wood^(^ ^29^ ^)^ (1993)	Butter	2·00	5·84	0·01	0·02
Almendingen^(^ ^31^ ^)^ (1995)	Butter	0·50	1·33	0·16	0·35
Aro^(^ ^30^ ^)^ (1997)	Run-in	0·80	2·29	0·14	0·04
	Run-in	0·80	2·29	0·37	0·27
Tholstrup^(^ ^24^ ^)^ (1998)	Danish milk	0·12	0·44	0·10	0·09
	Milk fat	1·66	6·18	0·26	0·18
Lichtenstein^(^ ^20^ ^)^ (2003)	Butter	1·00	3·1	0·57	0·43
	Butter	1·00	2·35	0·28	0·36
Biong^(^ ^26^ ^)^ (2004)	Cheese	0·68	1·53	0·10	0·11
Desroches^(^ ^28^ ^)^ (2005)	Control	0·25	0·83	− 0·41	− 0·19
	CLA	1·69	5·52	− 0·02	0·08
Tholstrup^(^ ^10^ ^)^ (2006)	Control	0·32	1·17	0·27	0·29
	Vaccenic acid	2·14	7·54	0·24	0·19
Tricon^(^ ^27^ ^)^ (2006)	Control	0·27	0·78	− 0·03	− 0·03
	CLA	2·10	6·34	0·18	0·09
Chardigny^(^ ^3^ ^)^ (2008)	R-TFA	4·19	10·03	0·07	0·03
	R-TFA	4·19	8·82	0·10	0·07
Motard-Bélanger^(^ ^4^ ^)^ (2008)	Moderate	1·45	5·4	0·23	0·47
	High	3·50	13·21	0·57	0·79
Malpuech-Brugère^(^ ^25^ ^)^ (2010)	L0	0·61	1·42	0·05	0·05
	L4	0·87	1·98	− 0·12	0·00
	L9	2·42	5·38	0·02	0·11
Lacroix^(^ ^11^ ^)^ (2012)	R-TFA	1·40	3·54	0·31	0·30

% EI, percentage of energy intake; CLA, conjugated linoleic acid; R-TFA, ruminant *trans-*fatty acid; L0, experimental dairy fat containing 2·9 g R-TFA for 100 g fatty acids; L4, experimental dairy fat containing 4·1 g R-TFA for 100 g fatty acids; L9, experimental dairy fat containing 12·2 g R-TFA for 100 g fatty acids.

* Experimental group, as named in the original articles.

### Relationship between the doses of total ruminant-trans-18 : 1 and changes in the ratio of total cholesterol and HDL-cholesterol (Δ_TC:HDL-C_) or changes in the ratio of LDL-cholesterol and HDL-cholesterol (Δ_LDL-C:HDL-C_)

**Fig. 2:**
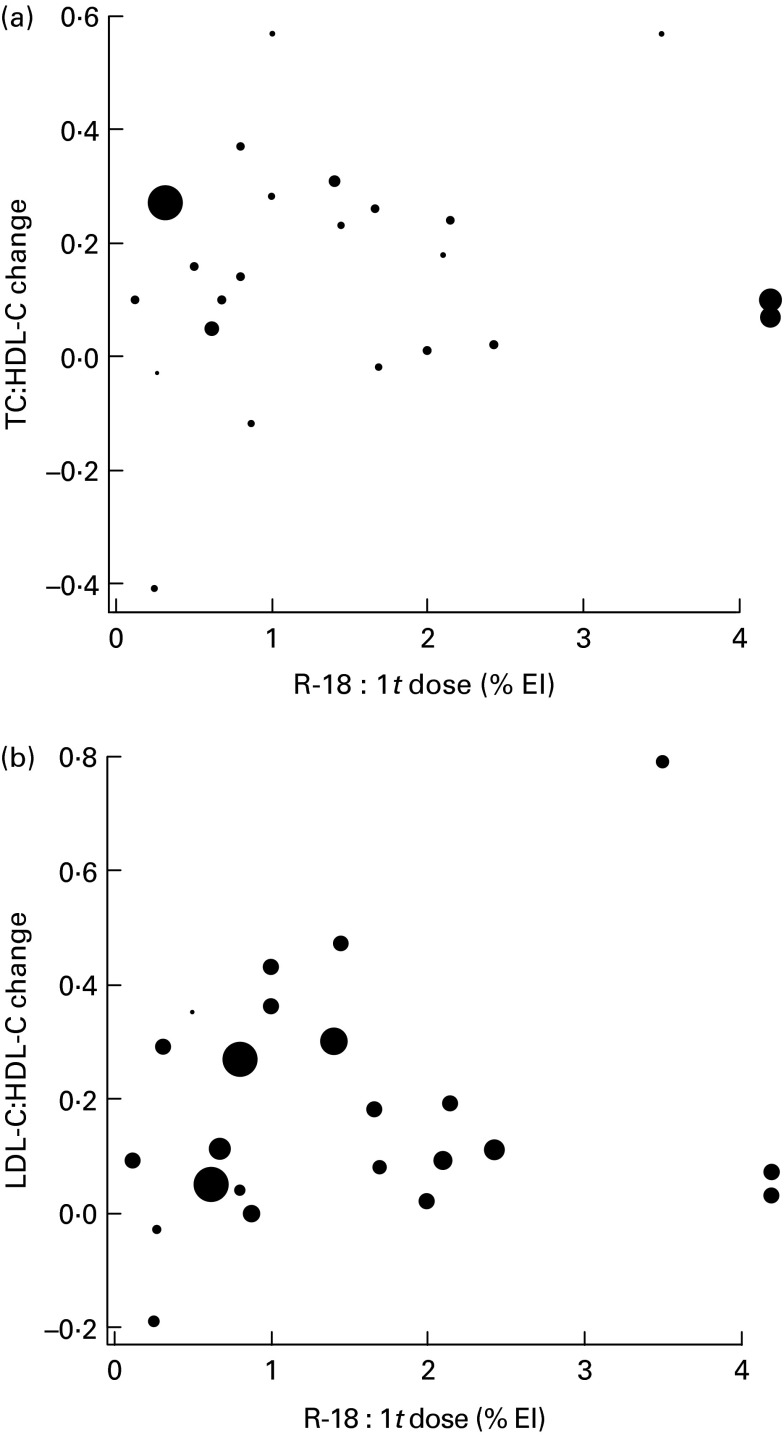
Univariate random-effects meta-regression analysis. Mean changes in the lipid ratios of total cholesterol (TC):HDL-cholesterol (HDL-C) (a) and LDL-cholesterol (LDL-C):HDL-C (b) are plotted against doses of ruminant-18 : 1 *trans*-fatty acids (R-18:1*t*; percentage of daily energy intake (% EI)) in the diet. The area of each circle is inversely proportional to the variance of the mean change in the ratios.

The univariate meta-regression analysis showed no significant association between the doses of total R-18 : 1*t* and either Δ_TC:HDL-C_ or Δ_LDL-C:HDL-C_. The slopes of both meta-regressions were not significantly different from 0 (*P*= 0·72, CI − 0·1064, 0·0737 and *P*= 0·77, CI − 0·1154, 0·1547, respectively). The intercept of both meta-regressions was positive but not significantly different from 0 (Fig. 2(a) and (b)). Using the case diagnostics of the metafor package, three among the twenty-three groups of subjects were detected as most influential on the results. To address whether these three data points generated most of the effect, we performed the analysis after exclusion of these study groups. The final result was not affected (data not shown). Finally, funnel plots assessing potential publication bias suggested the absence of literature publication bias for both outcomes (see online supplementary Fig. S1(A) and (B)).

### Exploration of potential modifiers influencing changes in the ratio of total cholesterol and HDL-cholesterol (Δ_TC:HDL-C_) or changes in the ratio of LDL-cholesterol and HDL-cholesterol (Δ_LDL-C:HDL-C_)

The backward stepwise PLS multivariate regression was further used to explore the confounding factors that might influence the changes in the ratio of TC:HDL-C. The variable of interest total R-18 : 1*t* was eliminated along with the following other variables, from the less to the most important: TC; HDL-C; SFA; LDL-C; MUFA; TAG; age; BMI; FAT; PROT; EI; total R-18 : 1*t*. The remaining significant independent variables were CARB and PUFA for both response variables Δ_TC:HDL-C_ and Δ_LDL-C:HDL-C_.

The quality of adjustment of the final model was *R* ^2^ 0·48 for the response variable Δ_TC:HDL-C_ and *R* ^2^ 0·31 for the response variable Δ_LDL-C:HDL-C_. For both response variables, the coefficients of the independent variables CARB and PUFA were positive and negative, respectively, suggesting that CARB and PUFA contributed, respectively, to an increase and a decrease in both Δ_TC:HDL-C_ and Δ_LDL-C:HDL-C_ variables.

## Discussion

The impact of dietary TFA on cardiovascular risk factors has been under debate since the Mensink & Katan^(^ ^1^ ^)^ report published in 1990. While the deleterious effect of the intake of IP-TFA is unanimously accepted, the question remains unclear regarding the intake of R-TFA. The consumption of IP-TFA in animal models has been shown to induce pro-inflammatory responses, hepatic steatosis and atherogenic lipoprotein profiles^(^ ^32^ ^,^ ^33^ ^)^. On the contrary, consistent data from the literature suggest that vaccenic acid may limit inflammation in obese and dyslipidaemic JCR:LA-cp rats^(^ ^34^ ^)^ and substantially improve atherogenic lipid profiles^(^ ^7^ ^)^.

The results obtained from intervention studies have been difficult to interpret and thus from which to draw a definitive conclusion. Inconsistencies in human data may be due to a lack of statistical power, or to differences either in the type of subjects who have been studied and/or in the doses of TFA used and/or the control diets. Also, with regard to the various sources of TFA supplementation, other components from the dairy matrix may influence such R-TFA effects. The literature suggests that the unique combination of nutrients and bioactive components may be responsible for the neutral/beneficial impact on CVD health.

The current main hypothesis argues that the null association of R-TFA with CVD or CHD risk may be due to the low level of R-TFA commonly consumed^(^ ^14^ ^,^ ^35^ ^)^. The present meta-regression analysis aims to shed light on this specific point, by increasing statistical power and enhancing the precision of estimates across multiple modest-size trials.

We extracted all the available data from the scientific literature, dealing with the dose of R-TFA and blood lipid indicators such as LDL-C and HDL-C concentrations in randomised intervention trials. This implies high-controlled studies, especially in terms of dietary intakes. In the statistical analysis, we considered the major confounders with respect to the group and the diet, including specific subclasses of dietary fat composition such as saturated, monounsaturated and polyunsaturated fats.

The present results showed that R-TFA intake levels up to 4·19 % EI, have no significant impact on the change in the ratios of either TC:HDL-C or LDL-C:HDL-C. The adjustment for multiple variables confirmed our findings.

Food and health authorities from various countries have estimated an R-TFA daily intake ranging from 0·5 to 1 % EI^(^ ^36^ ^,^ ^37^ ^)^. Previously, Jakobsen *et al.* ^(^ ^38^ ^)^ reported similar data among the European Danish population, which is considered as a representative of a wide range of consumers (1 % EI at the 90th percentile). The present meta-regression analysis included trials with high R-TFA intake levels, reaching up to 4·19 % EI, which could be considered as the non-observable adverse effects level. We also included trials in which low levels of R-TFA intake were tested. Recent literature has pointed to the beneficial effect of R-TFA at low intake levels compared with no effect at higher intake levels. The authors showed an inverse association between the intake of R-TFA and the change in body weight with a plateau above an R-TFA intake of 0·4 % EI^(^ ^17^ ^)^.

Our challenge was to investigate, based on the available literature, whether increased doses of R-TFA increase the risk of CVD. In the present meta-analysis, we included thirteen randomised clinical trials yielding twenty-three independent experimental groups. Both univariate and multivariate regression analyses were used, and we found no relationship between R-TFA intake levels and changes in the ratios of TC:HDL-C and LDL-C:HDL-C. To maximise the amount of data, we deliberately considered from the carefully selected trials specific arms of subjects who were eligible, with each group of volunteers acting as his/her own control. We then studied the difference in the parameters of interest, before and after the intervention period. However, we did not have the details on the previous diet, which may have influenced blood lipid levels at baseline. To limit possible bias, we confirmed homogeneity between groups at the beginning of the intervention period for both outcomes (TC:HDL-C and LDL-C:HDL-C). We also cannot exclude residual effects such as enrolling a subject in a trial, which may explain the positive, though not significant, intercept with the *y*-axis that we observed.

Our findings strongly suggest that at current levels of intake, R-TFA have no adverse effects on either of the well-recognised CVD risk markers TC:HDL-C or LDL-C:HDL-C ratio. Within the large range of R-TFA doses (0·12–4·19 % EI), we did not have graphical evidence that a threshold exists.

To consider potential confounders is of utmost importance. As a result, we used PLS multivariate regression to explore which confounding factors might influence the changes in the ratios of TC:HDL-C and LDL-C:HDL-C. The PLS multivariate regression analysis confirmed that the dose of R-TFA did not induce significant changes in any of the cholesterol indicators (TC:HDL-C and LDL-C:HDL-C). In line with the literature^(^ ^39^ ^,^ ^40^ ^)^, our findings also suggested that dietary CARB intake might increase the TC:HDL-C ratio, while PUFA intake might decrease it. However, the selection criteria of the articles were focused on R-TFA intakes rather than CARB or PUFA intakes, and the relevance and interpretation of such additional results are of limited value. Furthermore, recent literature strongly suggests that various PUFA may have different effects on cardiovascular outcomes^(^ ^41^ ^)^. Thus, it would be of great interest to consider *n*-3 and *n*-6 PUFA as separate variables. The literature also indicates that the effects of CARB intakes on CVD risk may vary depending on the quality of CARB^(^ ^42^ ^)^. Unfortunately, detailed data were not available and the present results on the potential influence of all PUFA as well as all CARB might suffer from a lack of nutritional relevance. It could have been interesting to also include dietary cholesterol intake as a confounding factor, but we had 40 % missing data for this variable and the strength of the present meta-analysis would have severely suffered.

While elevated plasma LDL-C concentrations are a potent risk factor for CVD, several other metabolic factors contribute to the aetiology of CVD. Elevated postprandial lipaemia, chronic inflammation, lipoprotein oxidation, impaired fibrinolytic activity, insulin resistance and endothelial dysfunction as well as increased blood pressure are very likely to contribute to the risk of CVD. Thus, data relating R-TFA to the risk of CVD based solely on variations in blood cholesterol markers need to be interpreted with caution. To date, the effects of R-TFA intake on those new metabolic markers have not been sufficiently investigated and are not well established. It will be of great interest in the future to perform further meta-analyses assessing the impact of specific R-TFA on a cluster of other cardiometabolic risk factors.

Some other limitations of the present meta-analysis should be considered. First, previous work has shown that R-TFA intake may affect men and women differently^(^ ^3^ ^,^ ^13^ ^)^, but owing to a lack of available data we were unable in the end to include this variable in our final model. Second, some change-from-baseline standard deviation data were missing and then imputed by using the coefficient correlation method referenced in the Cochrane Handbook. Although previous literature reported that standard deviation imputation did not alter the conclusion of the meta-analysis^(^ ^43^ ^,^ ^44^ ^)^, these calculated values may induce a bias. Third, we assessed the effect of total R-18 : 1*t* that represents approximately 80 % of the total R-TFA. Other R-TFA isomers may contribute to the overall impact of R-TFA intake on metabolism. However, besides R-*trans*-18 : 1 fatty acids, a well-recognised R-TFA isomer is palmitoleic acid (16 : 1*t n*-7), whose plasma levels have been recently associated with an improved metabolic profile^(^ ^45^ ^)^. Finally, most studies included in the present review had short-term follow-ups, from which we cannot draw a conclusion on the cumulative effect of R-TFA intake. In this regard, cohort studies can be helpful, and a recent meta-analysis of prospective studies has shown that R-TFA intake was not related to the risk of CHD^(^ ^14^ ^)^.

As we chose to focus on the effect of TFA from ruminant sources, our method did not allow comparison with IP-TFA intake. However, the literature on effects of IP-TFA on health has been consistent, and several studies have suggested that higher doses of IP-TFA are associated with increased risk of CVD^(^ ^46^ ^,^ ^47^ ^)^. Some other studies have demonstrated that IP-TFA might be pro-inflammatory and a higher intake of IP-TFA is associated with higher levels of circulating biomarkers of systemic inflammation^(^ ^48^ ^,^ ^49^ ^)^.

### Conclusion

In summary, while adverse cardiometabolic effects of IP-TFA are well established, data from the present meta-regression analysis of existing randomised controlled trials indicate that R-TFA intake levels do not substantially influence the changes in the ratios of TC:HDL-C and LDL-C:HDL-C in healthy adults. We included observations related to TFA from natural sources only, where doses of R-TFA were clearly documented. The present meta-analysis provides new data with respect to R-TFA intake levels, and has shed light on this specific question that remained unclear. Although more intervention trials are warranted to draw a definitive conclusion, the present meta-analysis strongly suggests that R-TFA at current levels of intake have no harmful effects on two well-recognised CVD risk markers. With respect to the conflicting debate on the topic of TFA intake and CVD risk, our findings support the belief that discrimination between TFA from the two sources (IP-TFA *v*. R-TFA) may be considered for further dietary guidelines.

## Supplementary material

To view supplementary material for this article, please visit http://dx.doi.org/10.1017/S0007114514002578

## References

[1] Mensink R & Katan MB (1990) Effect of dietary *trans* fatty acids on high density and low density lipoprotein cholesterol levels in healthy subjects. New Engl J Med 323, 439–445. 2374566 10.1056/NEJM199008163230703

[2] Willett WC, Stampfer MJ, Manson JE, et al. (1993) Intake of *trans* fatty acids and risk of coronary heart disease among women. Lancet 341, 581–585. 8094827 10.1016/0140-6736(93)90350-p

[3] Chardigny JM, Destaillats F, Malpuech-Brugère C, et al. (2008) Do *trans* fatty acids from industrially produced sources and from natural sources have the same effect on cardiovascular disease risk factors in healthy subjects? Results of the *trans* Fatty Acids Collaboration (TRANSFACT) study. Am J Clin Nutr 87, 558–566. 18326592 10.1093/ajcn/87.3.558

[4] Motard-Bélanger A, Charest A, Grenier G, et al. (2008) Study on the effect of *trans* fatty acids from ruminants on blood lipids and other risk factors for cardiovascular disease. Am J Clin Nutr 87, 593–599. 18326596 10.1093/ajcn/87.3.593

[5] Stender S, Astrup A & Dyerberg J (2012) A trans European Union difference in the decline in *trans* fatty acids in popular foods: a market basket investigation. BMJ Open 2, . 10.1136/bmjopen-2012-000859 PMC3467660 22986123

[6] Wang Y, Ruth MR, Goruk SD, et al. (2008) *Trans*-11 vaccenic acid dietary supplementation induces hypolipidemic effects in JCR:LA-cp rats. J Nutr 138, 2117–2122. 18936207 10.3945/jn.108.091009

[7] Wang Y, Jacome-Sosa MM, Ruth MR, et al. (2009) *Trans*-11 vaccenic acid reduces hepatic lipogenesis and chylomicron secretion in JCR:LA-cp rats. J Nutr 139, 2049–2054. 19759243 10.3945/jn.109.109488

[8] Mitchell PL, Langille MA, Currie DL, et al. (2005) Effect of conjugated linoleic acid isomers on lipoproteins and atherosclerosis in the Syrian Golden hamster. Biochim Biophys Acta 1734, 269–276. 15919237 10.1016/j.bbalip.2005.04.007

[9] Lock AL, Horne CA, Bauman DE, et al. (2005) Butter naturally enriched in conjugated linoleic acid and vaccenic acid alters tissue fatty acids and improves the plasma lipoprotein profile in cholesterol-fed hamsters. J Nutr 135, 1934–1939. 16046719 10.1093/jn/135.8.1934

[10] Tholstrup T, Raff M, Basu S, et al. (2006) Effects of butter high in ruminant *trans* and monounsaturated fatty acids on lipoproteins, incorporation of fatty acids into lipid classes, plasma C reactive protein, oxidative stress, hemostatic variables, and insulin in healthy young men. Am J Clin Nutr 83, 237–243. 16469980 10.1093/ajcn/83.2.237

[11] Lacroix E, Charest A, Cyr A, et al. (2012) Randomized controlled study of the effect of a butter naturally enriched in *trans* fatty acids on blood lipids in healthy women. Am J Clin Nutr 95, 318–325. 22205319 10.3945/ajcn.111.023408 PMC3260067

[12] Mensik RP, Zock PL, Kester AD, et al. (2003) Effects of dietary fatty acids and carbohydrates on the ratio of serum to HDL cholesterol and on serum lipids and apolipoproteins: a meta-analysis of 60 controlled trials. Am J Clin Nutr 77, 1146–1155. 12716665 10.1093/ajcn/77.5.1146

[13] Jakobsen MU, Overvad K, Dyerberg J, et al. (2008) Intake of ruminant *trans* fatty acids and risk of coronary heart diseases. Int J Epidemiol 37, 173–182. 18077475 10.1093/ije/dym243

[14] Bendsen NT, Christensen R, Bartels EM, et al. (2011) Consumption of industrial and ruminant *trans* fatty acids and risk of coronary heart disease: a systematic review and meta-analysis of cohort studies. Eur J Clin Nutr 65, 773–783. 21427742 10.1038/ejcn.2011.34

[15] Laake I, Pedersen JI, Selmer R, et al. (2012) A prospective study of intake of *trans*-fatty acids from ruminant fat, partially hydrogenated vegetable oils, and marine oils and mortality from CVD. Br J Nutr 108, 743–754. 22059639 10.1017/S0007114511005897

[16] Brouwer I, Wanders AJ & Katan MB (2013) *Trans* fatty acids and cardiovascular health: research completed? Eur J Clin Nutr 67, 541–547. 23531781 10.1038/ejcn.2013.43

[17] Hansen CP, Berentzen TL, Halkjær J, et al. (2012) Intake of ruminant *trans* fatty acids and changes in body weight and waist circumference. Eur J Clin Nutr 66, 1104–1109. 22805493 10.1038/ejcn.2012.87

[18] Codex Alimentarius Commission Codex Guidelines on Nutrition Labelling. CAC/GL2-1985. Chapter 2, definition 2.10; Adopted 1985. Revisions 1993 and 2011. Amendment 2003, 2006, 2009, 2010, 2012 and 2013. Annex adopted 2011 and revised 2013.

[19] Precht D & Molkentin J (1995) *Trans* fatty acids: implications for health, analytical methods, incidence in edible fats and intake (a review). Nahrung 39, 343–374. 8569844 10.1002/food.19950390503

[20] Lichtenstein AH, Erkkilä AT, Lamarche B, et al. (2003) Influence of hydrogenated fat and butter on CVD risk factors: remnant-like particles, glucose and insulin, blood pressure and C-reactive protein. Atherosclerosis 171, 97–107. 14642411 10.1016/j.atherosclerosis.2003.07.005

[21] Lewington S, Whitlock G, Clarke R, et al. (2007) Blood cholesterol and vascular mortality by age, sex, and blood pressure: a meta-analysis of individual data from 61 prospective studies with 55,000 vascular deaths. Lancet 370, 1829–1839. 18061058 10.1016/S0140-6736(07)61778-4

[22] Higgins JP & Green S (2008) Cochrane Collaboration. Cochrane Handbook for Systematic Reviews of Interventions. Chichester/Hoboken, NJ: Wiley-Blackwell.

[23] Viechtbauer W (2010) Conducting meta-analyses in R with the metafor package. J Stat Softw 36, issue 3.

[24] Tholstrup T, Sandström B, Hermansen JE, et al. (1998) Effect of modified dairy fat on postprandial and fasting plasma lipids and lipoproteins in healthy young men. Lipids 33, 11–21. 9470169 10.1007/s11745-998-0175-0

[25] Malpuech-Brugère C, Mouriot J, Boue-Vaysse C, et al. (2010) Differential impact of milk fatty acid profiles on cardiovascular risk biomarkers in healthy men and women. Eur J Clin Nutr 64, 752–759. 20485306 10.1038/ejcn.2010.73

[26] Biong AS, Müller H, Seljeflot I, et al. (2004) A comparison of the effects of cheese and butter on serum lipids, haemostatic variables and homocysteine. Br J Nutr 92, 791–797. 15533268 10.1079/bjn20041257

[27] Tricon S, Burdge GC, Jones EL, et al. (2006) Effects of dairy products naturally enriched with *cis*-9, *trans*-11 conjugated linoleic acid on the blood lipid profile in healthy middle-aged men. Am J Clin Nutr 83, 744–753. 16600923 10.1093/ajcn/83.4.744

[28] Desroches S, Chouinard PY, Galibois I, et al. (2005) Lack of effect of dietary conjugated linoleic acids naturally incorporated into butter on the lipid profile and body composition of overweight and obese men. Am J Clin Nutr 82, 309–319. 16087973 10.1093/ajcn.82.2.309

[29] Wood R, Kubena K, O'Brien B, et al. (1993) Effect of butter, mono- and polyunsaturated fatty acid-enriched butter, *trans* fatty acid margarine, and zero *trans* fatty acid margarine on serum lipids and lipoproteins in healthy men. J Lipid Res 34, 1–11. 8445333

[30] Aro A, Jauhiainen M, Partanen R, et al. (1997) Stearic acid, *trans* fatty acids, and dairy fat: effects on serum and lipoprotein lipids, apolipoproteins, lipoprotein(a), and lipid transfer proteins in healthy subjects. Am J Clin Nutr 65, 1419–1426. 9129471 10.1093/ajcn/65.5.1419

[31] Almendingen K, Jordal O, Kierulf P, et al. (1995) Effects of partially hydrogenated fish oil, partially hydrogenated soybean oil, and butter on serum lipoproteins and Lp[a] in men. J Lipid Res 36, 1370–1384. 7666013

[32] Dhibi M, Brahmi F, Mnari A, et al. (2011) The intake of high fat diet with different *trans* fatty acid levels differentially induces oxidative stress and non alcoholic fatty liver disease (NAFLD) in rats. Nutr Metab 23, 65. 10.1186/1743-7075-8-65 PMC3192664 21943357

[33] Kraft J, Spiltoir JI, Salter AM, et al. (2011) Differential effects of the *trans*-18 : 1 isomer profile of partially hydrogenated vegetable oils on cholesterol and lipoprotein metabolism in male F1B hamsters. J Nutr 141, 1819–1826. 21880955 10.3945/jn.111.143776

[34] Blewett HJ, Gerdung CA, Ruth MR, et al. (2009) Vaccenic acid favourably alters immune function in obese JCR:LA-cp rats. Br J Nutr 102, 526–536. 19216829 10.1017/S0007114509231722

[35] Weggemans RM, Rudrum M & Trautwein EA (2004) Intake of ruminant versus industrial *trans* fatty acids and risk of coronary heart disease – what is the evidence? Eur J Lipid Sci Technol 106, 390–397.

[36] US Food and Drug Administration & Center for Food and Safety and Applied Nutrition (2003) Food labeling: *trans* fatty acids in nutrition. Federal Register 68, 41433–414506. 12856667

[37] French Agency for Food Safety (2009) Opinion of February 20th, 2009 regarding the estimation of *trans* fatty acid intake in the French population. Request 2007-SA-0220.

[38] Jakobsen MU, Bysted A, Andersen NL, et al. (2006) Intake of ruminant *trans* fatty acids in the Danish population aged 1–80 years. Eur J Clin Nutr 60, 312–318. 16234830 10.1038/sj.ejcn.1602316

[39] Jakobsen MU, Dethlefsen C, Joensen AM, et al. (2010) Intake of carbohydrates compared with intake of saturated fatty acids and risk of myocardial infarction: importance of the glycemic index. Am J Clin Nutr 91, 1764–1768. 20375186 10.3945/ajcn.2009.29099

[40] Astrup A, Dyerberg J, Elwood P, et al. (2011) The role of reducing intakes of saturated fats in the prevention of cardiovascular disease: where does the evidence stand in 2010? Am J Clin Nutr 93, 684–688. 21270379 10.3945/ajcn.110.004622 PMC3138219

[41] Ramsden CE, Zamora D, Leelarthaepin B, et al. (2013) Use of dietary linoleic acid for secondary prevention of coronary heart disease and death: evaluation of recovered data from the Sydney Diet Heart Study and updated meta-analysis. BMJ 346, e8707. 23386268 10.1136/bmj.e8707 PMC4688426

[42] Jakobsen MU, O'Reilly EJ, Heitmann BL, et al. (2009) Major types of dietary fat and risk of coronary heart disease: a pooled analysis of 11 cohort studies. Am J Clin Nutr 89, 1425–1432. 19211817 10.3945/ajcn.2008.27124 PMC2676998

[43] Thiessen Philbrook H, Barrowman N & Garg AX (2007) Imputing variance estimates do not alter the conclusions of a meta-analysis with continuous outcomes: a case study of changes in renal function after living kidney donation. J Clin Epidemiol 60, 228–240. 17292016 10.1016/j.jclinepi.2006.06.018

[44] Furukawa TA, Barbui C, Cipriani A, et al. (2006) Imputing missing standard deviations in meta-analyses can provide accurate results. J Clin Epidemiol 59, 7–10. 16360555 10.1016/j.jclinepi.2005.06.006

[45] Mozaffarian D, Cao H, King IB, et al. (2010) Circulating palmitoleic acid and risk of metabolic abnormalities and new-onset diabetes. Am J Clin Nutr 92, 1350–1358. 20943795 10.3945/ajcn.110.003970 PMC2980960

[46] Lichtenstein AH, Ausman LM, Jalbert SM, et al. (1999) Effects of different forms of dietary hydrogenated fats on serum lipoprotein cholesterol levels. N Engl J Med 340, 1933–1940. 10379016 10.1056/NEJM199906243402501

[47] Mauger JF, Lichtenstein AH, Ausman LM, et al. (2003) Effect of different forms of dietary hydrogenated fats on LDL particle size. Am J Clin Nut 78, 370–375. 10.1093/ajcn/78.3.370 12936917

[48] Baer DJ, Judd JT, Clevidence BA, et al. (2004) Dietary fatty acids affect plasma markers of inflammation in healthy men fed controlled diets: a randomized crossover study. Am J Clin Nutr 79, 969–973. 15159225 10.1093/ajcn/79.6.969

[49] Mozaffarian D, Pischon T, Hankinson SE, et al. (2004) Dietary intake of *trans* fatty acids and systemic inflammation in women. Am J Clin Nutr 79, 606–612. 15051604 10.1093/ajcn/79.4.606 PMC1282449

